# Preliminary effects and feasibility of online interactive Baduanjin exercise in adults with overweight and obesity: a pilot randomized controlled trial

**DOI:** 10.3389/fendo.2025.1529705

**Published:** 2025-04-16

**Authors:** Yan Yu, Jinpeng Wu, Tongtong Wu, Genghang Chen, Xueyin Chen, Shaonan Liu, Yu Chen, Lihong Yang, Xinfeng Guo

**Affiliations:** ^1^ Second Clinical Medical College of Guangzhou University of Chinese Medicine, Guangzhou, China; ^2^ Institute of Biomedical Engineering, Chinese Academy of Medical Sciences & Peking Union Medical College, Tianjin, China; ^3^ The Second Affiliated Hospital of Guangzhou University of Chinese Medicine (Guangdong Provincial Hospital of Chinese Medicine), Guangzhou, China; ^4^ State Key Laboratory of Dampness Syndrome of Chinese Medicine, The Second Affiliated Hospital of Guangzhou University of Chinese Medicine, Guangzhou, China

**Keywords:** Baduanjin, obesity, overweight, randomized controlled trial, pilot study

## Abstract

**Background:**

Overweight and obesity are chronic conditions with severe health implications, demanding effective and sustainable management strategies. The escalated Baduanjin, an adapted form of traditional Baduanjin, is proposed as a targeted intervention for individuals with overweight and obesity, offering a potentially effective and accessible approach to weight management and overall health improvement.

**Objectives:**

This pilot study aimed to assess the preliminary effects and feasibility of the online interactive escalated Baduanjin exercise program, with a focus on participant engagement and acceptance.

**Methods:**

A 12-week pilot randomized controlled trial was conducted in Guangzhou, China, from June 30 to November 15, 2023, involving 50 participants with overweight and obesity. Participants were randomly allocated to the intervention group (n=26) or the control group (n=24). The intervention group received three 60-minute online interactive escalated Baduanjin exercises per week for 12 weeks, in addition to health education. The control group received only health education, delivered in three sessions over the 12-week period. The primary outcome was the change in body mass index (BMI) from baseline to week 12. Secondary outcomes included changes in waist circumference, body weight, blood glucose, lipid levels, blood pressure, quality of life, and dampness scale scores. Feasibility was assessed by participant adherence to the required intervention, and adverse events were recorded throughout the study period.

**Results:**

Compared to the control group, the intervention group exhibited a reduction in BMI (mean ± SD: -0.54 ± 1.67 vs. -0.13 ± 0.81), body weight, diastolic blood pressure, fasting blood glucose, and triglyceride levels over the 12 weeks, though this difference was not statistically significant. However, the intervention group demonstrated significant improvements in several health parameters, including waist circumference, fatigue scale-14 (FS-14), general anxiety disorder (GAD-7), patient health questionnaire-9 items (PHQ-9), Pittsburgh sleep quality index (PSQI), and dampness scale scores. Adherence to the intervention was high, with 82% (41/50) of participants completing the trial, and no serious adverse events were reported.

**Conclusion:**

The preliminary effects and feasibility of the online interactive escalated Baduanjin for adults with overweight and obesity have been demonstrated, highlighting its potential multifaceted health benefits and high adherence.

**Clinical Trial Registration:**

https://www.chictr.org.cn, identifier ChiCTR2300072981.

## 1 Introduction

Overweight and obesity have become prioritized global public health challenges. The World Obesity Federation released *World Obesity Atlas 2024*, which indicates that the prevalence of overweight and obesity is increasing globally, from 42% of the world’s adults in 2020 to over 54% by 2035. This trend is accompanied by a significant economic burden, with obesity-related costs rising from 2.4% of global GDP in 2020 to an estimated $4.32 trillion by 2035 (1). Similarly, in China, a 2.8% annual growth rate in the number of adults with a high body mass index (BMI) from 2020-2035 was reported, and nearly 6.0 billion adults may be affected by 2035 (1). The impact of overweight and obesity is increasing the risk of chronic diseases and cardiovascular mortality while also placing a significant and long-term burden on healthcare systems and national economies (2–5), which highlights the urgency for effective and accessible interventions.

Given the limitations of conventional medical approaches, there is growing interest in exploring cultural or non-conventional strategies, such as traditional Chinese exercises, which involve slow movement, incorporating meditation and breathing (6–8), to complement existing interventions (7, 8). Among these, our previous evidence map of traditional Chinese exercises has noted that compared to other traditional Chinese exercises, Baduanjin shows more prominent effects on obesity and other metabolic disorders (9). In addition, findings from a systematic review also demonstrated that compared to daily activities, lecture or acupoint embedding, engaging in Baduanjin exercise for 8 to 24 weeks, with a weekly duration of 4-7 times each lasting 40-90 minutes, significantly reduced weight (−3.69 [95% confidence interval (CI), −4.97 to −2.40]), BMI (−5.42 [95% CI, −6.56 to −4.28]) and waist circumference (WC) (−1.36 [95% CI, −1.76 to −0.96]) (10). Despite being a safer weight-loss option for individuals with overweight and obesity who struggle with high-impact exercise, Baduanjin faces several challenges: its relatively low intensity, inadequate evaluation methods that fail to capture its full range of clinical benefits, and the limitations of centralized or in-person exercise sessions. These factors restrict its accessibility and scalability and make long-term adherence difficult, thereby hindering optimal weight-loss outcomes (11–13).

Recognizing these limitations, we propose an adapted approach to the escalated Baduanjin, which integrates traditional elements with enhancements to provide a potentially more effective approach to weight management and even multifaceted clinical improvement. The escalated Baduanjin consists of two phases: the first phase follows the traditional Baduanjin, while the second phase develops a strengthened version. Compared with the traditional Baduanjin, the strengthened version focuses more on joint mobility, incorporating more joints, and extending the duration of static stretches. These modifications are designed to improve flexibility, strength, and coordination, offering a more effective and sustainable weight management strategy. Moreover, previous studies have shown that commute distance to research sites is one of the main barriers for individuals with overweight and obesity to participate in clinical studies (14). To address this, we developed the escalated Baduanjin as an online interactive exercise program, allowing participants to exercise at home under the guidance of a coach. This approach leverages online platforms to overcome distance barriers, offering both convenience and guidance, to improve adherence.

Therefore, a pilot randomized controlled trial (RCT) was conducted to evaluate the multifaceted clinical effects and the feasibility of an online interactive escalated Baduanjin program. We planned to test the hypotheses, including: 1) The escalated Baduanjin, with increased stretching angles, prolonged pauses, and optimized frequency, will yield significant clinical benefits. 2) Beyond weight and metabolic improvements, the escalated Baduanjin will demonstrate multifaceted improvements, including dampness syndrome, fatigue, insomnia, and emotional well-being, areas often neglected in prior research. 3) The online interactive format will maintain comparable efficacy to centralized and in-person exercises while achieving higher adherence due to its convenience and accessibility.

## 2 Methods

### 2.1 Study design

This was a pilot RCT in which participants were randomly allocated to the escalated Baduanjin exercise group or the control group. The design of a pilot RCT is appropriate for evaluating the preliminary efficacy and feasibility of the escalated Baduanjin exercise program before scaling up to a full-scale study. The trial followed the principles of the Declaration of Helsinki, adhered to the reporting guidelines for pilot RCTs (**Supplementary Appendix 1**: CONSORT checklist) (15), was approved by the Ethics Committee at the Guangdong Provincial Hospital of Chinese Medicine (No. YF2023–109-01) and was registered on the Chinese clinical trial registry (chictr.org.cn, No. ChiCTR2300072981) on 30 June 2023.

To ensure participant confidentiality, all personal information was anonymized and stored securely. Participants were assigned unique identification codes, and only authorized personnel had access to the data. Potential exercise-related risks were addressed through a comprehensive safety protocol, which included pre-exercise health screenings, real-time monitoring during online sessions, and immediate follow-up for any reported adverse events. Informed consent was obtained from all participants.

### 2.2 Setting and participants

This study was conducted at Guangdong Hospital of Chinese Medicine between 30 June 2023 and 15 November 2023. Promotional posters, including the inclusion criteria for the trial, were distributed in outpatient clinics and on social media. Participants are strictly screened by personnel before participating in the study to ensure that those who meet the inclusion criteria are admitted to the study.

The eligible participants were individuals who (1) were overweight or obese (BMI≥24 kg/m^2^ or WC ≥90 cm [men] or ≥85 cm [women] for adults in China) (16); (2) presented dampness syndrome, that is the score of dampness syndrome scale ≥20 (17); (3) were aged 18–65 years; (4) could practice the escalated Baduanjin exercise for 3 months; and (5) provided signed informed consent. Dampness syndrome, a traditional Chinese medicine concept, is a cluster of symptoms primarily characterized by fatigue, drowsiness, heavy cumbersome limbs, poor appetite, abdominal distention, etc. (17, 18) It was assessed using a validated 30-item scale with a total score of 120 to measure these physical and digestive symptoms. Higher scores indicate more severe dampness syndrome and a score of ≥20 was used as the cutoff to define the presence of dampness syndrome (17).

The exclusion criteria were as follows: (1) pregnant women; (2) comorbid any physical disabilities or serious diseases that are not suitable for the intervention (e.g., neurologic diseases, musculoskeletal disorders or mental system diseases); (3) engaging in regular exercise or other weight loss interventions, such as acupuncture, embedding, drugs, etc.; and (4) participation in other interventional clinical trials.

### 2.3 Randomization, allocation concealment and blinding

Eligible participants were randomized into either the escalated Baduanjin exercise group or the control group. The randomization sequence was generated via SPSS 18.0 software by an independent researcher who was not involved in the study. The sequentially numbered, opaque sealed envelopes kept by independent personnel were used to conceal the randomization sequence. Participants were assigned to groups using these envelopes following the completion of all baseline assessments. Given the nature of the intervention, the participants and personnel involved in conducting the study could not be blinded, but the outcome assessors, laboratory technicians, data managers, and statisticians were blinded to the treatment allocations. These personnel must avoid interacting with the participants during the study period.

### 2.4 Interventions

#### 2.4.1 Control group

The control group was provided with health education sessions three times within 12 weeks after enrollment, with each session lasting 60 minutes. Health education was delivered by clinical experts online. Education topics included an overview of overweight and obesity, setting appropriate weight loss goals, and strategies for diet and exercise for weight loss. The attendance of health education sessions of both groups was verified through online time during the sessions. If the online time exceeded 45 minutes each time, it was indicated that the participants had completed the health education.

#### 2.4.2 Intervention group

In addition to receiving the same health education as the control group, participants in the intervention group were also provided with the escalated Baduanjin exercise, which was low to moderate-intensity aerobic exercise (19). The escalated Baduanjin exercise program was a 12-week exercise program, with three times and four repetitions of 60-minute online interactive exercises per week (7, 10, 20). The entire process of the escalated Baduanjin exercise was led by the coach and carried out in the form of online courses. A flexible exercise time was allowed. The participants could practice the required exercise sessions and time every week at any place at their convenience. Participants were required to follow along with teaching videos provided by the coach if they were unable to attend the online courses on time. They were also expected to submit their exercise videos which were evaluated by the researchers to ensure proper form and technique.

**Figure 1:**
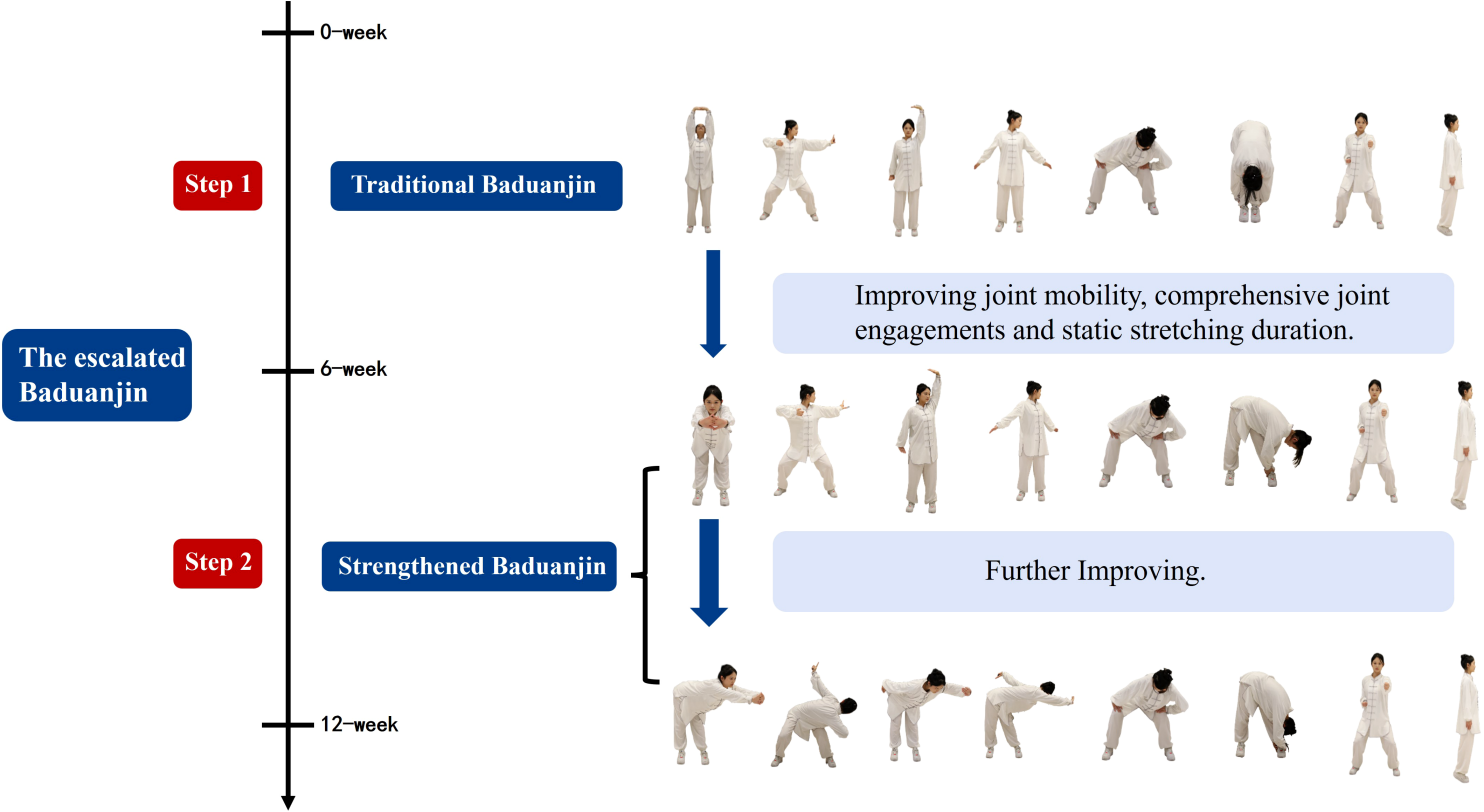
Graphical depiction of the escalated Baduanjin.

The escalated Baduanjin exercise program consists of two phases, gradually improving flexibility, strength, and coordination through step-by-step exercises to achieve multifaceted health benefits. The first phase lasted from weeks 1 to 5, and the participants practiced the traditional Baduanjin movements, which were issued by the General Administration of Sport of China (step 1). The second phase was from weeks 6 to 12, and the participants performed the strengthened Baduanjin exercise (step 2). The strengthened Baduanjin, based on the traditional Baduanjin, focused on increasing the amplitude and holding time of forward bending, backward bending, and turning movements, thereby enhancing joint mobility, comprehensive joint engagements and static stretching duration to enhance significant effects on health and fitness (**Figure 1**). Detailed descriptions of the escalated Baduanjin are provided in **Supplementary Appendix 2**. Progression criteria between phases were based on participants’ physical readiness and adherence to the exercise protocol. Sessions were tailored to accommodate participants with varying physical abilities through individualized coaching and modifications as needed.

Participants were encouraged to achieve at least 75% of the required exercise protocol; if not, they were considered low adherence. For participants who did not exercise according to the protocol, researchers and coaches reminded them to exercise and inquired about the reasons for their lack of exercise, while adopting a reward mechanism to provide motivational support. Given the influence of factors like diet, sleep patterns, and other lifestyle habits on overweight and obesity (21), all participants were required to maintain their usual daily habits and avoid any drastic habitual changes during the study period. They were also required not to engage in other weight loss treatments, such as acupuncture, thread embedding, or additional exercise, during the intervention period.

### 2.5 Data collection

Data, including demographic and clinical characteristics, were collected at baseline via questionnaires. Adherence was collected from self-reported home exercises (based on exercise videos provided by participants) and online attendance rates (each online time of more than 45 minutes was considered for completion).

All outcomes were collected at baseline and after the 12-week intervention. The primary outcome was the change from baseline in BMI which was calculated as BMI (kg/m^2^) = body weight (kg)/height (m^2^). The secondary outcomes included changes from baseline to week 12 in WC, body weight, glycated hemoglobin (HbAlc), fasting blood glucose (FBG), fasting insulin (FINS), insulin resistance index (HOMA-IR), triglyceride (TG), total cholesterol (TC), high-density lipoprotein cholesterol (HDL-C), low-density lipoprotein cholesterol (LDL-C) and blood pressure; fatigue scale-14 (FS-14), general anxiety disorder (GAD-7), patient health questionnaire-9 items (PHQ-9), the Pittsburgh sleep quality index (PSQI) and dampness scale score. Details of the outcome measurements are listed in **Supplementary Appendix 3**.

### 2.6 Adverse events

Adverse events are defined as any undesirable occurrence that participants endure during the trial period, regardless of whether it is associated with the intervention. Any adverse events reported by participants were recorded by researchers and the coach during outcome measurements and intervention contacts.

### 2.7 Statistical analysis

#### 2.7.1 Sample size estimation

Previous studies have shown that a minimum of 20 participants should be included in a pilot study as this seems to be the smallest amount that is reasonable from statistical modeling studies. However, if we wanted to estimate the main study’s standard deviation, we should probably seek a sample size of at least 50 (22, 23).

Our study is a pilot RCT to explore the preliminary effects and feasibility of an online interactive escalated Baduanjin exercise program for adults with overweight and obesity. The primary outcome of BMI is a continuous variable. Considering the precision and efficiency, the proposed total sample size is 50 participants.

#### 2.7.2 Analytical approach

Outcome measurements were analyzed via full analysis sets according to intention-to-treat (ITT) analysis. A per-protocol set (PPS) was employed for sensitivity analysis on the basis of the data from adherence to the intervention protocol and completion of data collection.

The Shapiro–Wilks test and histogram were used to test the normal distribution of continuous data. Continuous data conforming to a normal distribution were summarized as the mean and standard deviation, if not as the median and interquartile range. Independent t-test and Mann-Whitney U test were used for analyses. Categorical data are presented as counts and percentages and were analyzed by the chi-square test. In addition, mean imputation methods will be used as a sensitivity analysis to address the missing data.

All the statistical analyses were performed via SPSS 18.0 software. Statistical significance was set at a two-sided p-value of less than 0.05.

## 3 Results

**Figure 2:**
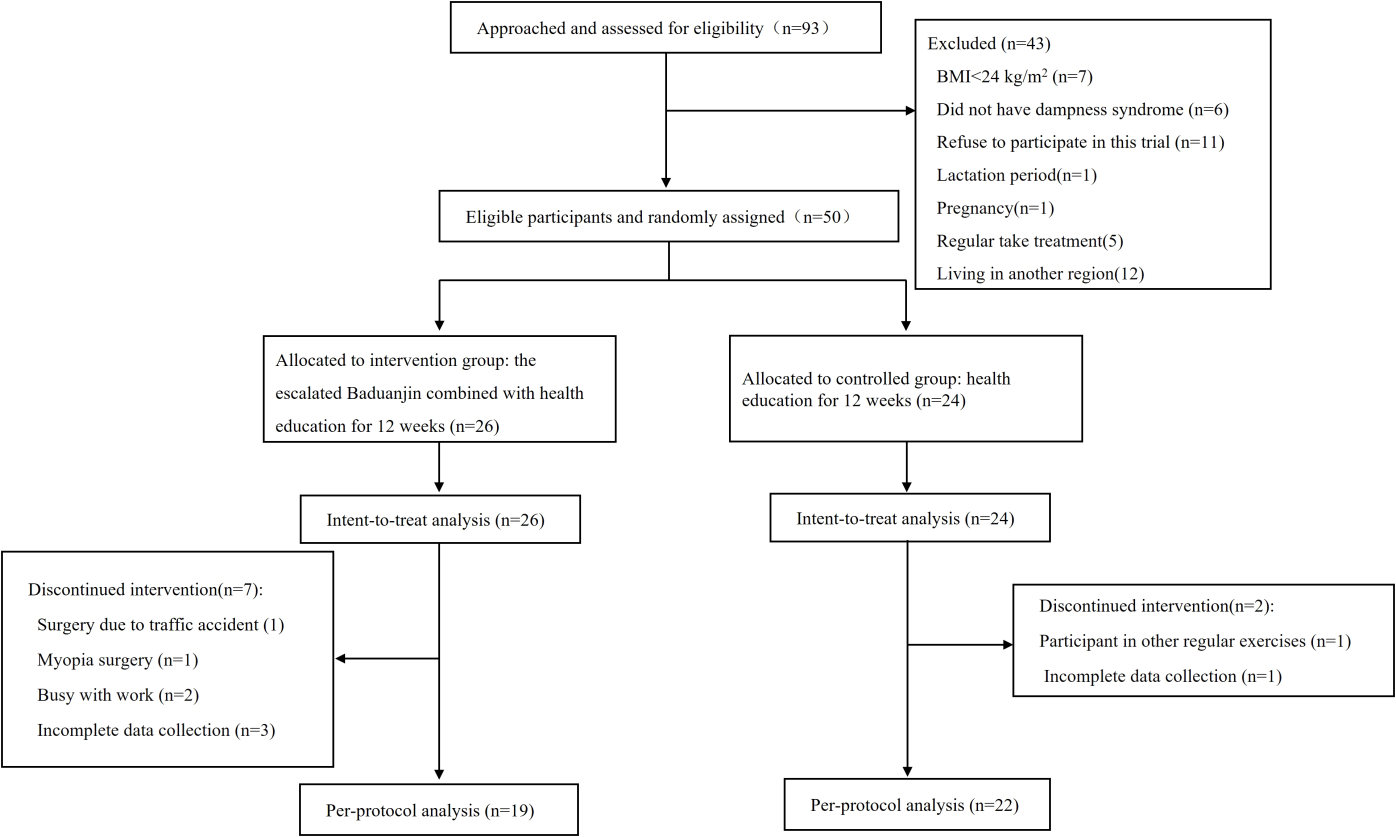
Flowchart of the pilot study.

A total of 93 individuals were screened at Guangdong Hospital of Chinese Medicine from June to July 2023. After those who were ineligible or refused to participate were excluded, a total of 50 eligible participants were enrolled, with 26 randomly assigned to the escalated Baduanjin exercise group and 24 to the control group. Five participants withdrew during the study period, with four from the intervention group because of car accidents (n=1), myopia surgery (n=1) and busy work (n=2). One participant in the control group withdrew due to a protocol violation, which involved participation in other exercises. Four participants (one from the control group and the other from the intervention group) did not complete the postintervention outcome measurements, resulting in missing data. Consequently, the PPS included 19 participants in the intervention group and 22 in the control group (**Figure 2**).

### 3.1 Baseline characteristics of the participants

The baseline characteristics of the participants are summarized in **Table 1**. Among all the participants, 78% were female and 22% were male, with a median age of 35.8 years. According to the WHO Asian BMI threshold, 54% of the participants were overweight (n=27), 46% were obese (n=23), and 92% were central obese. Additionally, 20% (n=10) of the participants had comorbidities, including polycystic ovary syndrome (n=4), nonalcoholic fatty liver disease (n=4), hyperuricemia (n=1), and hypertension (n=1). There were no significant differences between the two groups (*P* > 0.05).

**Table 1:** Baseline characteristics of participants.

Variables	Intervention Group (n=26)	Control Group (n=24)	*P*
Sex, n (%)			0.623
Male	5.0 (19.2)	6.0 (25.0)	
Female	21.0 (80.8)	18.0 (75.0)	
Age, year, $\bar{X}$ ± SD	33.6 ± 7.4	38.2 ± 9.3	0.060
Education, n (%)			0.928
<High school	2.0 (7.7)	0.0 (0.0)	
High school	3.0 (11.5)	4.0 (16.7)	
College	16.0 (61.5)	16.0 (66.7)	
>College	5.0 (19.2)	4.0 (16.7)	
Fertility Statue, n (%)			0.536
Without children	12.0 (46.2)	9.0 (37.5)	
With children	14.0 (53.8)	15.0 (62.5)	
Smoking status, n (%)			0.081
Nonsmoker	25.0 (96.2)	18.0 (75.0)	
Former smoker	1.0 (3.8)	3.0 (12.5)	
Current smoker	0.0 (0.0)	3.0 (12.5)	
Alcohol drinking status, n (%)			0.829
Nondrinker	11.0 (42.3)	8.0 (33.3)	
Former drinker	2.0 (7.7)	2.0 (8.3)	
Occasional drinker	13.0 (50)	14.0 (58.3)	
Physical Activity, n (%)			0.650
Inactive	8.0 (30.8)	6.0 (25.0)	
Occasional exercise (1-3 times/month)	18.0 (69.2)	6.0 (25.0)	
Eating status			
Total dishes, n (%)			0.585
Vegetarian diet	0.0 (0.0)	1.0 (4.2)	
Meat-based food	4.0 (15.4)	5.0 (20.8)	
Meat and vegetable balance	22.0 (84.6)	18.0 (75.0)	
Type of staple food, n (%)			0.728
Refined grains	13.0 (50.0)	13.0 (54.2)	
Coarse grains and tubers	2.0 (7.7)	2.0 (8.3)	
Refined grains + coarse grains and tubers	9.0 (34.6)	9.0 (37.5)	
No staple food	2.0 (7.7)	0.0 (0.0)	
Eating late-night meals, n (%)			0.374
3-6 times/week	0.0 (0.0)	3.0 (12.5)	
1-2 times/week	8.0 (30.8)	6.0 (25.0)	
≤3 times/month	7.0 (26.9)	7.0 (29.2)	
Avoid late-night meals	11.0 (42.3)	8.0 (33.3)	
Dietary habits, n (%)			0.278
Regular eating habits	17.0 (65.4)	19.0 (79.2)	
Irregular eating habits	9.0 (34.6)	5.0 (20.8)	
BMI, kg/m^2^	27.8 (25.6, 29.6)	27.0 (25.3, 29.2)	0.778
Waist circumference, cm, $\bar{X}$ ± SD	93.3 ± 7.0	94.3 ± 9.0	0.668
Duration of overweight/obesity, year, M (IQR)	5.0 (2.8, 10.0)	7.0 (3.3, 18.0)	0.418
Maximum weight, kg, M (IQR)	74.0 (70.0, 80.0)	75.0 (72.0, 88.3)	0.484
Medical history (regarding other conditions), n (%)			0.620
Yes	4.0 (15.4)	6.0 (25.0)	
No	22.0 (84.6)	18.0 (75.0)	
Family medical history (regarding overweight or obesity among close relatives), n (%)			0.963
Yes	15.0 (57.7)	14.0 (58.3)	
No	11.0 (42.3)	10.0 (41.7)	

### 3.2 Effects on primary outcome

**Table 2** presents the primary and secondary outcome results. Compared with the changes from baseline to week 12 in the control group, the intervention group showed an attenuation of the decrease in BMI (M ± SD: -0.54 ± 1.67 vs. -0.13 ± 0.81), but without significant difference (P=0.307).

**Table 2:** Primary and secondary outcomes.

	Baseline		Postintervention			Between-Group Difference	
	$\bar{X}$ ± SD or M (IQR)	P^*^	$\bar{X}$ ± SD or M (IQR)	*P^†^*	*P^‡^*	$\bar{X}$ ± SD or M (IQR)	*P^§^*
Primary outcome							
BMI, kg/m^2^		0.778			0.580		0.307
Intervention group	27.77 (25.62, 29.62)		26.74 (24.42, 29.40)	0.163		-0.54 ± 1.67	
Control group	27.04 (25.33, 29.23)		26.34 (25.42, 29.12)	0.466		-0.13 ± 0.81	
Secondary outcomes							
Waist circumference, cm		0.668			0.047		0.027
Intervention group	93.27 ± 7.03		90.38 ± 9.19	0.081		-2.23 ± 5.40	
Control group	94.25 ± 9.01		96.11 ± 8.98	0.162		1.93 ± 6.25	
Weight, kg		0.600			0.980		0.277
Intervention group	73.25 (69.48, 78.33)		70.7 (66.55, 79.23)	0.149		-1.42 ± 4.22	
Control group	72.50 (67.73, 79.00)		72.15 (67.03, 77.45)	0.515		-0.3 ± 2.12	
SBP, mmHg		0.207		0.051			0.897
Intervention group	117 (111.25, 123)		118.5 (111, 121)	0.505		1.15 ± 7.57	
Control group	119.5 (112.25, 127.75)		122.5 (115, 127.75)	0.794		0.73 ± 12.93	
DBP, mmHg		0.093		0.005			0.052
Intervention group	76.23 ± 10.24		72.5 (65.25, 81.5)	0.171		-2 (-8, 2.5)	
Control group	81.33 ± 10.82		80.5 (76.75, 92)	0.121		2 (-1, 5.5)	
FS-14		0.598			0.020		0.011
Intervention group	8.38 ± 3.51		4.5 (3.25, 7)	0.000		-3.1 ± 3.02	
Control group	8.83 ± 2.41		8.5 (4, 11)	0.186		-0.77 ± 2.65	
GAD-7		0.274			0.055		0.035
Intervention group	6 (2, 8.25)		2 (0, 5)	0.026		-2.2 ± 4.06	
Control group	3 (0.25, 7.75)		4 (2, 7)	0.581		0.45 ± 3.80	
PHQ-9		0.334		0.053			0.008
Intervention group	5.5 (2, 11.25)		2.5 (2, 5)	0.007		-2 (-6, -0.25)	
Control group	3 (1.25, 10.25)		4.5 (2.75, 8)	0.431		1 (-1.25, 3)	
PSQI		0.407			0.013		0.003
Intervention group	7.65 ± 4.09		5 (3.25, 6.75)	0.002		-2.5 (-4, -0.25)	
Control group	6.79 ± 3.08		6 (5, 9)	0.470		1 (-1, 2)	
FBG, mmol/l		0.808			0.347		0.219
Intervention group	5.16 (4.78, 5.55)		4.94 (4.45, 5.15)	0.02		-0.3 (-0.45, 0)	
Control group	5.08 (4.87, 5.41)		4.97 (4.79, 5.41)	0.13		-0.08 (-0.37, 0.07)	
HbAlc		0.598			0.537		0.441
Intervention group	5.6 (5.28, 5.85)		5.5 (5.3, 5.9)	0.341		0 (-0.2, 0.1)	
Control group	5.55 (5.3, 5.78)		5.45 (5.28, 5.65)	0.014		-0.1 (-0.2, 0)	
FINS, pmol/l		0.877			0.583		0.676
Intervention group	84.61 (53.69, 109.57)		80.81 (46.51, 105.17)	0.872		-0.99 (-20.73, 28.8)	
Control group	83.29 (56.79, 111.31)		80.09 (49.13, 132.97)	0.131		-7.37 (-24.56, 6.42)	
HOMA-IR		0.816			0.734		0.657
Intervention group	2.64 (1.79, 3.42)		2.26 (1.58, 3.24)	0.717		-0.04 (-0.82, 0.55)	
Control group	2.66 (1.78, 3.50)		2.46 (1.55, 4.32)	0.072		-0.30 (-0.70, 0.17)	
TG, mmol/l		0.244			0.097		0.906
Intervention group	1.24 (0.86, 1.52)		0.93 (0.72, 1.42)	0.409		-0.09 (-0.31, 0.2)	
Control group	1.39 (0.90, 2.07)		1.09 (0.94, 1.91)	0.237		-0.05 (-0.37, 0.12)	
TC, mmol/l		0.039			0.409		0.108
Intervention group	4.76 (4.18, 4.99)		4.77 ± 0.69	0.673		-0.09 (-0.26, 0.51)	
Control group	5.24 (4.24, 5.78)		4.99 ± 0.934	0.044		-0.24 (-0.54, 0.17)	
HDL-C, mmol/l		0.977			0.901		0.162
Intervention group	1.31 (1.17, 1.44)		1.39 ± 0.23	0.024		0.1 (0.02, 0.18)	
Control group	1.24 (1.14, 1.49)		1.38 ± 0.28	0.258		0.03 (-0.05, 0.13)	
LDL-C, mmol/l		0.513			0.610		0.747
Intervention group	3.24 ± 0.55		3.30 ± 0.68	0.433		0.07 ± 0.35	
Control group	3.39 ± 0.99		3.42 ± 0.85	0.866		0.02 ± 0.52	
Dampness syndrome scale		0.573			0.001		0.000
Intervention group	41.5 (37.75, 63.75)		26.5 (20.25, 31)	0.000		-16 (-30, -8.75)	
Control group	44.5 (32, 53.25)		40 (30, 47.25)	0.079		-4 (-9.5, 1.25)	

BMI, body mass index; SBP, systolic blood pressure; DBP, diastolic blood pressure; FS-14, fatigue scale; GAD-7, general anxiety disorder; PHQ-9, patient health questionnaire-9 items; PSQI, Pittsburgh sleep quality index; FBG, fasting blood glucose; HbAlc, Glycated hemoglobin; FINS, fasting insulin; HOMA-IR, insulin resistance index; TG, triglyceride; TC, total cholesterol; HDL-C, high-density lipoprotein cholesterol; LDL-C, low-density lipoprotein cholesterol.

P^*^ for comparing baseline data between the intervention group and control group; *P^†^* for comparing the changes in the intervention group or control group from baseline to the 12-week intervention; *P^‡^* for comparing the postintervention data between the intervention group and control group; *P^§^* for comparing changes from baseline to the 12-week intervention between the two groups.

### 3.3 Effects on secondary outcomes

Compared with the control group, the intervention group demonstrated statistically significant reductions in WC (M ± SD: -2.23 ± 5.40 vs. 1.93 ± 6.25), FS-14 score (M ± SD: -3.1 ± 3.02 vs. -0.77 ± 2.65), GAD-7 score (M ± SD: -2.2 ± 4.06 vs. 0.45 ± 3.80), PHQ-9 score (M(IQR): -2(-6, -0.25) vs. 1(-1.25, 3)), PSQI score (M(IQR): -2.5(-4, -0.25) vs. 1(-1, 2)), and dampness scale score (M(IQR): -16(-30, -8.75) vs. -4(-9.5, 1.25)). No significant differences in other secondary outcomes were detected between the two groups, both in postintervention values and 12-week changes, but weight, diastolic blood pressure, FBG, and TG were significantly lower in the intervention group than in the control group.

### 3.4 Sensitivity analyses

The results of the two sensitivity analyses involving the FAS after mean imputation and the PPS were consistent with those of the primary analysis, which used ITT analysis. The results are shown in **Table 3**.

**Table 3:** Sensitivity analysis.

	Baseline		Postintervention			Between-Group Difference	
	$\bar{X}$ ± SD or M (IQR)	P^*^	$\bar{X}$ ± SD or M (IQR)	*P^†^*	*P^‡^*	$\bar{X}$ ± SD or M (IQR)	*P^§^*
Mean imputation							
BMI, kg/m^2^		0.778			0.697		0.854
Intervention group	27.77 (25.62, 29.62)		27.72 (24.96, 28.60)	0.534		-0.01 (-0.79, 0.55)	
Control group	27.04 (25.33, 29.23)		27.13 (25.61, 29.06)	0.306		-0.04 (-0.75, 0.37)	
Waist circumference, cm		0.668			0.047		0.022
Intervention group	93.27 ± 7.03		91.07 ± 8.12	0.041		-2.20 ± 5.20	
Control group	94.25 ± 9.01		95.89 ± 8.62	0.211		1.64 ± 6.23	
Dampness syndrome scale		0.573			0.002		0.000
Intervention group	41.5 (37.75, 63.75)		30 (24, 34.38)	0.000		-16 (-32, -8.46)	
Control group	44.5 (32, 53.25)		39 (30.75, 46.5)	0.083		-4 (-8.75, 1.75)	
Per-Protocol Set (PPS)							
BMI, kg/m^2^		0.778			0.774		0.703
Intervention group	27.77 (25.67, 29.62)		27.38 (24.58, 29.45)	0.312		-0.24 ± 0.99	
Control group	27.04 (25.33, 29.23)		26.34 (25.43, 29.12)	0.466		-0.13 ± 0.81	
Waist circumference, cm		0.668			0.081		0.044
Intervention group	93.27 ± 7.03		89 (84, 96)	0.142		-1.87 ± 5.30	
Control group	94.25 ± 9.01		95.5 (88.75, 102)	0.162		1.93 ± 6.25	
Dampness syndrome scale		0.573			0.002		0.000
Intervention group	41.5 (37.75, 63.75)		27 (20, 31)	0.000		-16 (-32, -8)	
Control group	44.5 (32, 53.25)		40 (30, 47.25)	0.079		-4 (-9.5, 1.25)	

BMI, body mass index; P^*^ for comparing baseline data between the intervention group and control group; *P^†^* for comparing the changes in the intervention group or control group from baseline to the 12-week intervention; *P^‡^* for comparing the postintervention data between the intervention group and control group; *P^§^* for comparing changes from baseline to the 12-week intervention between the two groups.

### 3.5 Feasibility of the pilot study

**Figure 3:**
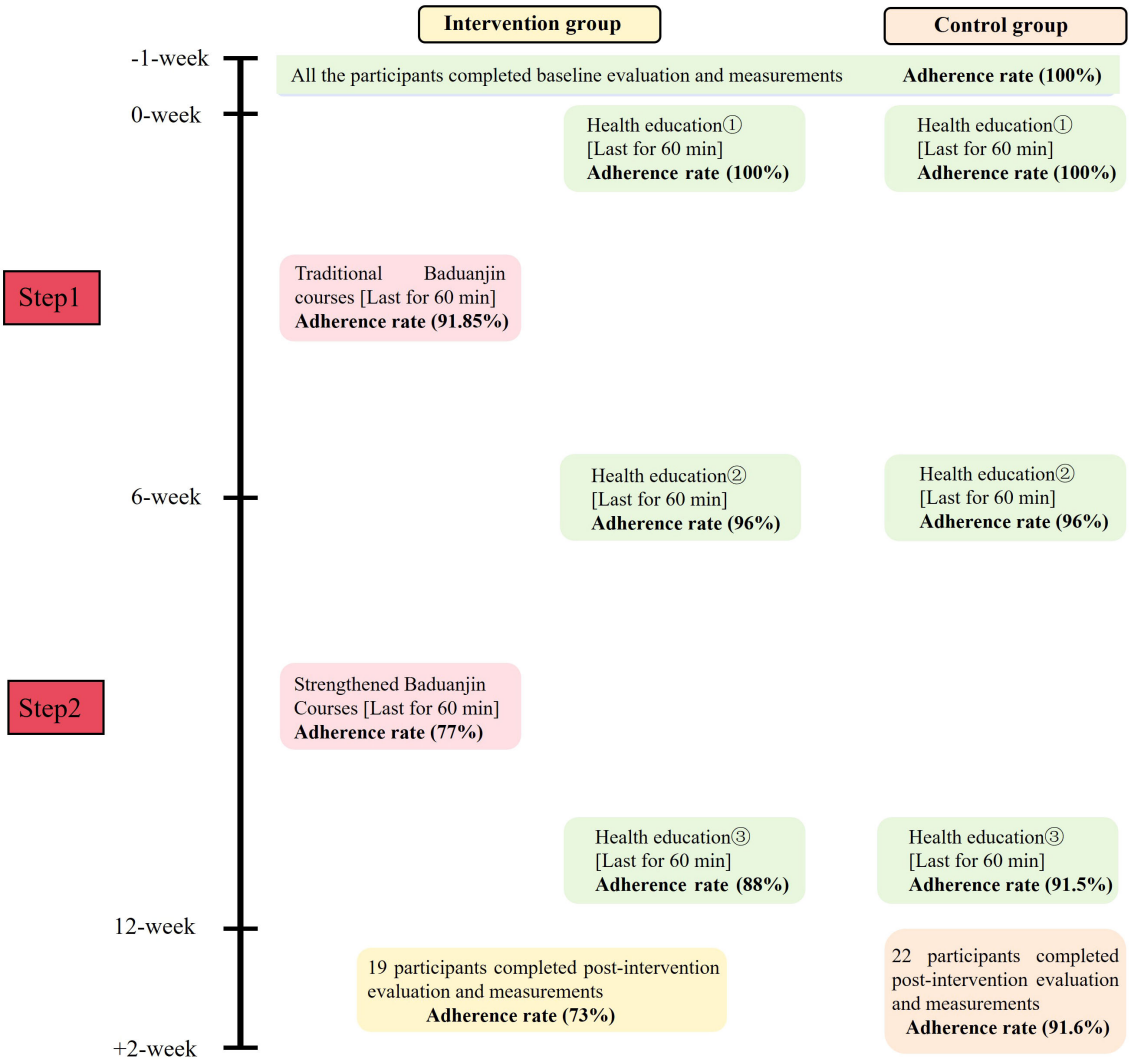
Intervention schedule and adherence rate.

Note: Attend rate in the intervention group = (n1/p+n2/p+n3/p…+n26/p)/26; Attend rate in the control group = (n1/p+n2/p+n3/p…+n24/p)/24; p: the number of all the courses per stage; n1/p-n26/p: represents the attendance rate of 26 participants in the intervention group.

We recruited a sufficient sample for the pilot study within a month through social media and outpatient clinics via posters, with the highest number of recruits coming from the outpatient clinic. The fidelity results indicated that the online interactive escalated Baduanjin exercise is feasible, with high adherence. However, adherence gradually declined as the study progressed. For the escalated Baduanjin exercise group, the average adherence rate was 91.85% in weeks 1-5 for the traditional Baduanjin exercise, and 77% in weeks 6-12 for the strengthened Baduanjin. For health education, the average adherence rate in the control group was higher than that in the intervention group. All participants completed baseline data collection, and the average adherence rate for outcome data collection postintervention was 73% in the intervention group and 91.6% in the control group. The intervention schedule and adherence rate are presented in **Figure 3**.

### 3.6 Adverse events

Among the 26 participants in the intervention group, there were 3 cases of muscle soreness and 4 cases of fatigue after exercise during the 12-week, which were all relieved after rest without affecting exercise. No other adverse events related to the intervention were observed during the intervention period.

## 4 Discussion

In this pilot study, a 12-week online interactive escalated Baduanjin exercise program achieved significant multifaceted benefits in waist circumference, fatigue, anxiety, depression, sleep quality and dampness syndrome for adults with overweight and obesity. In contrast with the findings in most related trials (7, 10), the changes in BMI were modest and did not reach statistical significance. However, as a pilot study, the online interactive format of the exercise is feasible and participants showed high adherence to the required exercises. Importantly, no severe adverse events were reported during the study period.

### 4.1 Multifaceted effects of the online interactive escalated Baduanjin exercise program

Waist circumference is a sensitive indicator of amassed visceral and ectopic fat accumulation, providing a more specific assessment and prediction of central obesity and its associated health risks of cardiovascular and metabolic morbidity and mortality (24–26). A reduction in WC is considered more important than weight loss in multidisciplinary obesity management (24). Our findings observed a significant reduction in WC among overweight and obesity, which is consistent with previous studies indicating that aerobic exercise, including escalated Baduanjin, can effectively reduce WC and visceral adipose tissue without necessarily leading to substantial weight loss (27–29).

The significant reduction in WC, despite the modest and non-significant changes in BMI, can be outlined across several factors. This pilot study indicated that the escalated Baduanjin movements focus more on the abdominal region. Evidence also suggests that Baduanjin can elevate the abdominal temperature (30), improve muscle glucose absorption and tolerance capacity to regulate insulin resistance (31), meanwhile effectively trigger mechanisms for fat oxidation energy and exert anti-inflammatory effects to regulate fat accumulation, modulate lipid metabolism and effectively reduce visceral fat (10, 32). In our study, 92% of participants were central obese, characterized by larger baseline WC measurements and greater accumulation of fat around the abdomen which provided greater scope for improvement. In contrast, the baseline BMI values of the participants were relatively low, limiting the potential for notable substantial changes in BMI over the short exercise period. Furthermore, the intensity and the exercise duration of the escalated Baduanjin may have been insufficient to induce significant changes in the participants who were predominantly aged 20-40 years.

Overweight and obesity are complex health issues that affect not only body weight but also involve multiple aspects of health, including metabolism, cardiovascular health, and mental well-being. In our study, the escalated Baduanjin has also shown significant effects on fatigue, anxiety, depression, and sleep. Aerobic exercise can regulate the hypothalamic-pituitary-adrenal axis, increase the levels of norepinephrine and release endogenous opioids to alleviate anxiety and depression (33). Meanwhile, it can also increase the oxygen consumption of the body, promote the release of brain endorphins, and increase melatonin levels, thus effectively improving fatigue and sleep quality (34–36).

### 4.2 Feasibility analysis and future research directions

#### 4.2.1 Recruitment strategies

Recruitment is a critical aspect of clinical trials, impacting both clinical practice and the extent to which the trial question has been addressed (37, 38). While recruitment through hospital outpatient clinics was relatively rapid in our study, such methods may prove insufficient for full-scale studies requiring larger sample sizes. To address this limitation, it is essential to explore alternative recruitment strategies, such as partnerships with healthcare providers and community organizations, and leveraging more social media platforms to expand recruitment channels and enhance the representativeness of the study population for full-scale studies (39).

#### 4.2.2 Target population

The escalated Baduanjin exercise program demonstrated significant efficacy in reducing WC, highlighting its potential benefits for individuals with central obesity. This finding underscores the need for further investigation into the mechanisms behind the favorable effects of the escalated Baduanjin on WC and visceral fat. Furthermore, participants in the pilot study were predominantly female, aged between 20-40 years, primarily comprising students and individuals with stable employment. These participants often lead fast-paced lifestyles and face significant family and work pressures, which may impact their adherence to low to moderate exercise. To consider these factors, it would be advantageous to include middle-aged and elderly individuals with central obesity who lead more leisurely lifestyles in future full-scale studies.

#### 4.2.3 Combining online and offline sessions

In our study, the online exercise format attracted most of the participants and demonstrated high adherence rates, likely due to its flexibility, allowing participants to exercise at any time and place. However, this approach also faced challenges, such as unclear video quality, which hindered both coaches and participants from accurately observing movements, as well as difficulties encountered by elderly participants in operating the necessary devices. These limitations highlight the potential for a hybrid approach that combines the convenience of online sessions with the benefits of centralized exercise and in-person guidance. For example, incorporating offline exercise sessions every two weeks or monthly could provide additional support and motivation, improve the evaluation of escalated Baduanjin exercise quality and efficacy, and maintain the flexibility and accessibility of the online format.

#### 4.2.4 Exercise quality

In addition to the format of online interactive exercise affecting the exercise quality, the lack of objective measurement of exercise intensity and duration, as well as the coach adherence, can also impact the quality and effectiveness of the exercise. Wearable devices to monitor key metrics such as heart rate and session duration, can provide real-time data on exercise intensity and duration, allowing for a more accurate assessment of adherence and exercise quality. Additionally, coach adherence to whether exercises are prescribed, tailored, or demonstrated in accordance with protocols is essential for maintaining exercise quality (40). Coaches are necessary to use a written intervention manual containing an outline/script and checklist of critical topics to be covered. All the sessions should be audiotaped and reviewed with the coaches during weekly meetings, timely adjusting the exercise sessions to reinforce intervention fidelity, provide feedback for researchers and improve the exercise quality (41).

#### 4.2.5 Adherence

Adherence to the intervention significantly affects the feasibility of the entire trial. Compared to other studies that have implemented offline, centralized exercises for obesity management, our study demonstrated higher adherence rates (20), which could be attributed to the advantages of the online interactive exercise, such as flexibility, convenience, and accessibility. However, adherence declined in weeks 6-12 of the study compared to weeks 1-5. There could be several potential reasons for this decrease. The primary motivation of most participants was to lose weight. When the exercise outcomes do not meet participants’ expectations within the initial period, it may dampen their enthusiasm and motivation to continue the exercise. In addition, the intensity of the escalated Baduanjin might have been insufficient for younger participants who may require a more challenging exercise to achieve desired results. In response to these issues, we propose several measures to enhance adherence in the full-scale trial. First, providing comprehensive education to participants, covering both their understanding of overweight and obesity and their knowledge of Baduanjin, to help them establish reasonable weight loss goals and boost their awareness and motivation. Second, further optimizing the escalated Baduanjin program, including the intensity, frequency and duration. Third, implementing a two-week run-in period and conducting an adherence test before randomization to identify and retain participants who demonstrate good adherence to the study protocol before enrolling them in the formal trial.

### 4.3 Limitations

This pilot study had potential limitations to note. First, the pilot study population was mostly female, overweight and aged between 20 and 40 years. Full-scale studies should aim for a more balanced representation across gender, BMI categories, and age groups to enhance the generalizability of the findings. Second, the form of online interactive exercise tends to overestimate adherence and challenge exercise quality and supervision. The full-scale study can develop a combination of online and offline exercise, and objectively measure the quality of exercise through scales, bracelets, or wearable devices. Third, Unmeasured factors, such as changes in diet, sleep patterns, and other lifestyle behaviors, might have influenced the results. Future studies should incorporate comprehensive assessments of these factors to provide a more accurate evaluation of the intervention’s effects. Furthermore, future full-scale studies should explore comparisons between the escalated Baduanjin and other normal activities to better identify the specific advantages of the escalated Baduanjin intervention.

## 5 Conclusion

The pilot study indicates that the online interactive escalated Baduanjin exercise program for adults with overweight and obesity is effective and feasible, supporting our initial hypotheses. The escalated Baduanjin exercise program showed multifaceted benefits, including reducing waist circumference and improving anxiety, depression, sleep quality, fatigue, and dampness syndrome, as well as the online interactive format of this exercise, highlights high adherence.

The full-scale study warranted refinement of the issues identified during pilot study processes, and further investigation is warranted to elucidate the comprehensive impact on health outcomes.

## Data Availability

The original contributions presented in the study are included in the article/**Supplementary Material**. Further inquiries can be directed to the corresponding authors.
