# Supplementary material for: Preliminary effects and feasibility of online interactive Baduanjin exercise in adults with overweight and obesity: a pilot randomized controlled trial
Source: Front Endocrinol (Lausanne). 2025 Apr 16;16:1529705. doi: 10.3389/fendo.2025.1529705 (PMC12040658; doi:10.3389/fendo.2025.1529705)
Supplement: Supplementary file 1 [file Table1.docx]

**Appendix 1**

**
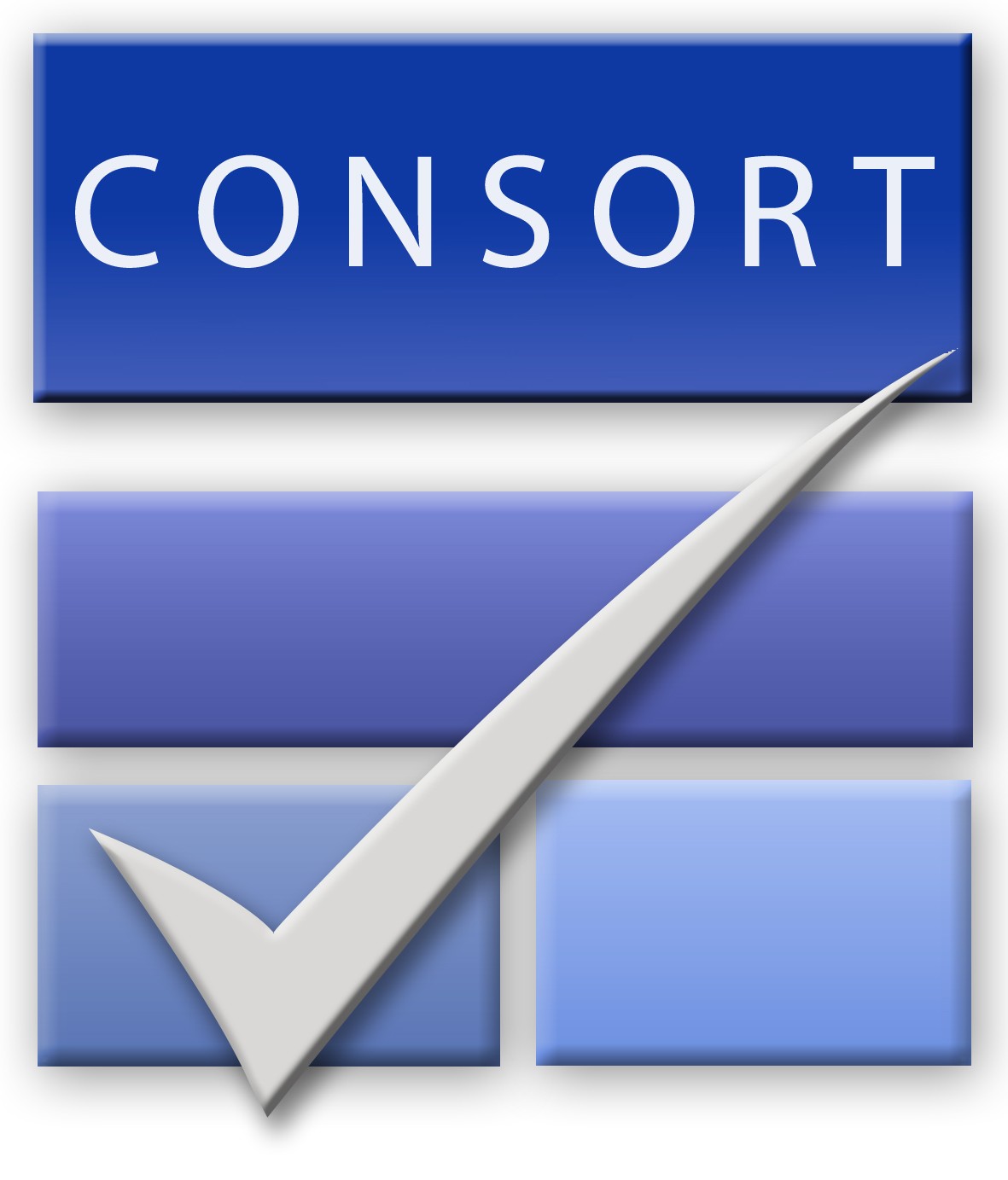
CONSORT 2010 checklist of information to include when reporting a pilot or feasibility trial***

| **Section/Topic** | **Item No** | **Checklist item** | **Reported on page No** |
| --- | --- | --- | --- |
| **Title and abstract** | | | |
|  | 1a | Identification as a pilot or feasibility randomised trial in the title | 1 |
|  | 1b | Structured summary of pilot trial design, methods, results, and conclusions (for specific guidance see CONSORT abstract extension for pilot trials) | 1-2 |
| **Introduction** | | | |
| Background and objectives | 2a | Scientific background and explanation of rationale for future definitive trial, and reasons for randomised pilot trial | 2 |
|  | 2b | Specific objectives or research questions for pilot trial | 2-3 |
| **Methods** | | | |
| Trial design | 3a | Description of pilot trial design (such as parallel, factorial) including allocation ratio | 3 |
|  | 3b | Important changes to methods after pilot trial commencement (such as eligibility criteria), with reasons | NA |
| Participants | 4a | Eligibility criteria for participants | 3 |
|  | 4b | Settings and locations where the data were collected | 3 |
|  | 4c | How participants were identified and consented | 3 & Figure 2 |
| Interventions | 5 | The interventions for each group with sufficient details to allow replication, including how and when they were actually administered | 3-4 & Figure 1 & Appendix 2 |
| Outcomes | 6a | Completely defined prespecified assessments or measurements to address each pilot trial objective specified in 2b, including how and when they were assessed | 4-5 & Appendix 3 |
|  | 6b | Any changes to pilot trial assessments or measurements after the pilot trial commenced, with reasons | NA |
|  | 6c | If applicable, prespecified criteria used to judge whether, or how, to proceed with future definitive trial | NA |
| Sample size | 7a | Rationale for numbers in the pilot trial | 5 |
|  | 7b | When applicable, explanation of any interim analyses and stopping guidelines | NA |
| Randomisation: |  |  |  |
| Sequence  generation | 8a | Method used to generate the random allocation sequence | 3 |
|  | 8b | Type of randomisation(s); details of any restriction (such as blocking and block size) | 3 |
| Allocation  concealment  mechanism | 9 | Mechanism used to implement the random allocation sequence (such as sequentially numbered containers), describing any steps taken to conceal the sequence until interventions were assigned | 3 |
| Implementation | 10 | Who generated the random allocation sequence, who enrolled participants, and who assigned participants to interventions | 3 |
| Blinding | 11a | If done, who was blinded after assignment to interventions (for example, participants, care providers, those assessing outcomes) and how | 3 |
|  | 11b | If relevant, description of the similarity of interventions | NA |
| Statistical methods | 12 | Methods used to address each pilot trial objective whether qualitative or quantitative | 5 |
| **Results** | | | |
| Participant flow (a diagram is strongly recommended) | 13a | For each group, the numbers of participants who were approached and/or assessed for eligibility, randomly assigned, received intended treatment, and were assessed for each objective | 5-6 & Figure 2 |
|  | 13b | For each group, losses and exclusions after randomisation, together with reasons | 5-6 & Figure 2 |
| Recruitment | 14a | Dates defining the periods of recruitment and follow-up | 5-6 & Figure 2 |
|  | 14b | Why the pilot trial ended or was stopped | NA |
| Baseline data | 15 | A table showing baseline demographic and clinical characteristics for each group | 5 & Table 1 |
| Numbers analysed | 16 | For each objective, number of participants (denominator) included in each analysis. If relevant, these numbers should be by randomised group | 5-7 & Figure 2 |
| Outcomes and estimation | 17 | For each objective, results including expressions of uncertainty (such as 95% confidence interval) for any  estimates. If relevant, these results should be by randomised group | 5-6 & Table 2 |
| Ancillary analyses | 18 | Results of any other analyses performed that could be used to inform the future definitive trial | 5-6 & Table 2 |
| Harms | 19 | All important harms or unintended effects in each group (for specific guidance see CONSORT for harms) | NA |
|  | 19a | If relevant, other important unintended consequences | NA |
| **Discussion** | | | |
| Limitations | 20 | Pilot trial limitations, addressing sources of potential bias and remaining uncertainty about feasibility | 11-12 |
| Generalisability | 21 | Generalisability (applicability) of pilot trial methods and findings to future definitive trial and other studies | 9-12 |
| Interpretation | 22 | Interpretation consistent with pilot trial objectives and findings, balancing potential benefits and harms, and  considering other relevant evidence | 5-12 |
|  | 22a | Implications for progression from pilot to future definitive trial, including any proposed amendments | 9-12 |
| **Other information** | | |  |
| Registration | 23 | Registration number for pilot trial and name of trial registry | 2 |
| Protocol | 24 | Where the pilot trial protocol can be accessed, if available | Register website |
| Funding | 25 | Sources of funding and other support (such as supply of drugs), role of funders | 12 |
|  | 26 | Ethical approval or approval by research review committee, confirmed with reference number | 3 |

Citation: Eldridge SM, Chan CL, Campbell MJ, Bond CM, Hopewell S, Thabane L, et al. CONSORT 2010 statement: extension to randomised pilot and feasibility trials. BMJ. 2016;355.

*We strongly recommend reading this statement in conjunction with the CONSORT 2010, extension to randomised pilot and feasibility trials, Explanation and Elaboration for important clarifications on all the items. If relevant, we also recommend reading CONSORT extensions for cluster randomised trials, non-inferiority and equivalence trials, non-pharmacological treatments, herbal interventions, and pragmatic trials. Additional extensions are forthcoming: for those and for up to date references relevant to this checklist, see [www.consort-statement.org](http://www.consort-statement.org).

**Appendix 2**

| **Appendix 2. The escalated Baduanjin exercise** | | |
| --- | --- | --- |
| **Section 1. Both hands hold up the heavens to balance the triple warmer** | | |
| **Traditional Baduanjin** | **Strengthened Baduanjin (First + Second levels)** | |
| 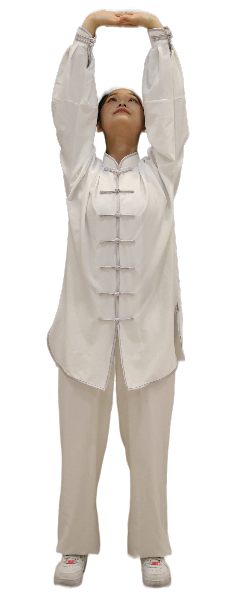  Stand with your feet shoulder-width apart. Cross your palms in front of your abdomen, palms facing up, and lift them upwards to your chest, internally rotate and raise your arms above your head, stretching as high as you comfortably can. Hold the stretch for a moment, then lower your arms. | 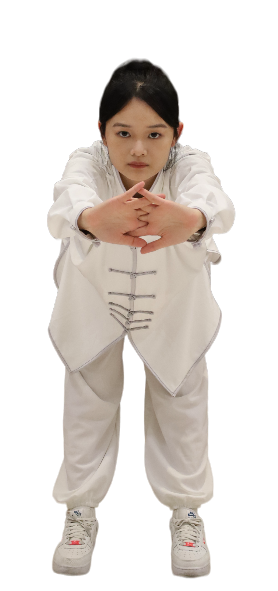  Repeat the movements of Baduanjin to raise and stretch both hands over the head as high as possible. Bend forward by 90° and hold the position for a breathing period, then bend back to the extreme and stay for a breathing period. Then return to the starting position. Repeat this movement several times. | 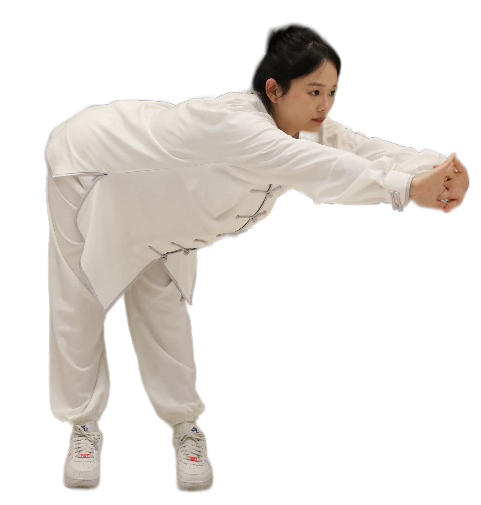  Repeat the movements of Baduanjin to raise and stretch both hands over the head as high as possible and hold this position. Keep the position of hips and turn the waist to the left for 45°, then bend to the left front for 90°, stay for a breathing period, and return to the starting position. Repeat this movement on the other side. |
| **Section 2. Drawing the Bow to Left and Right Shooting the hawk** | | |
| **Traditional Baduanjin** | **Strengthened Baduanjin (First + Second levels)** | |
| 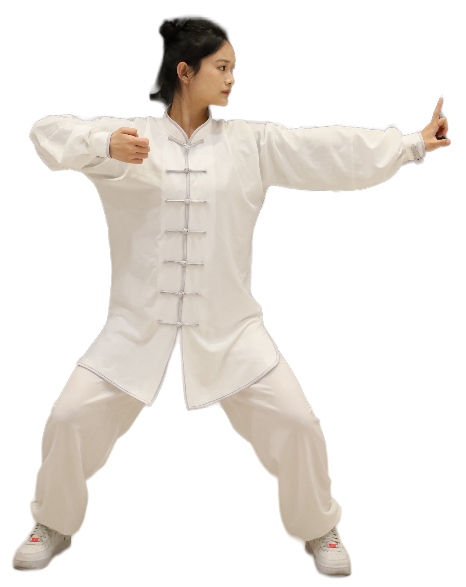  Stand with your feet wider than shoulder-width apart and your arms hanging loosely at your sides. Center of gravity move right, step out to the left, and squat into a horse step as you extend your left arm out with your pointer finger and thumb creating an "L" shape as far as possible. At the same time pull your right arm to the right side like holding the string with your right hand. Your right elbow is bent with your right and left arms in a straight line. Then return to the starting position. Repeat this movement on the other side. | 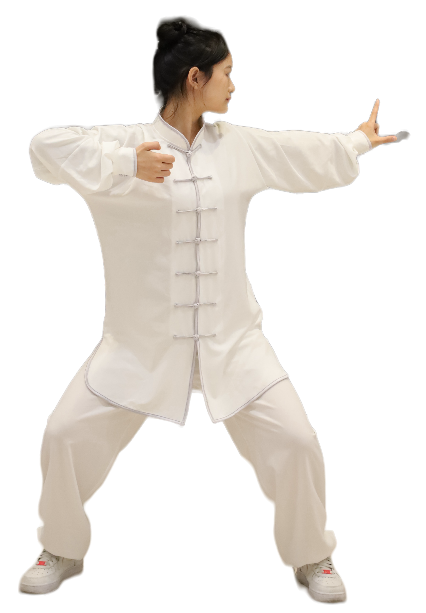  Repeat the movements of Baduanjin to draw the bow and squat into a horse step. Keep the position of hips and turn the waist to the left for 45°, stay for a breathing period, and return to the starting position. Repeat this movement on the other side. | 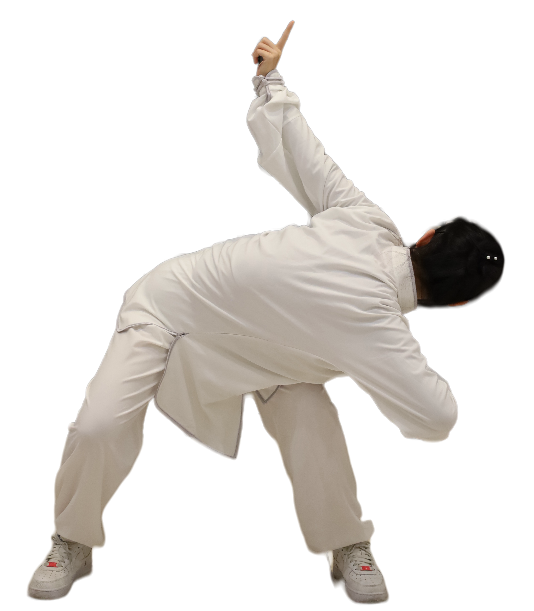  Repeat the movements of Baduanjin to draw the bow and squat into a horse step, Keep the position of hips and turn the waist to the left for 45°, staying for a breathing period. Then bend your torso to the left front while turning to the extreme left. Stretch your left palm upwards and backward, lower your right shoulder, and support your right elbow downward, drawing the bow with both hands. Focus your gaze on the tip of your left index finger, staying for a breathing period. Finally, return to the starting position and repeat this movement on the other side. |
| **Section 3. Separating Heaven and Earth to Balance the Spleen and Stomach** | | |
| **Traditional Baduanjin** | **Strengthened Baduanjin (First + Second levels)** | |
| 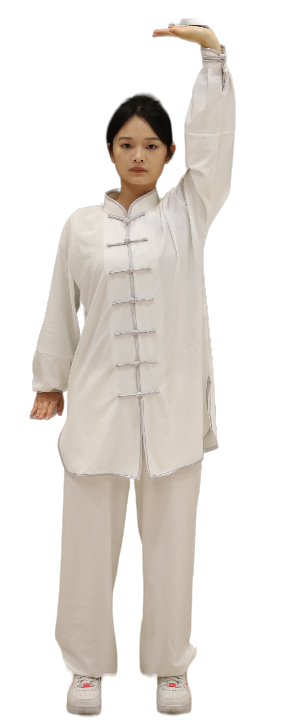  Stand with your feet shoulder-width apart and your arms at your sides. Raise one arm and lower the other, stretching as far as possible. Then return to the starting position. Repeat this movement on the other side. | 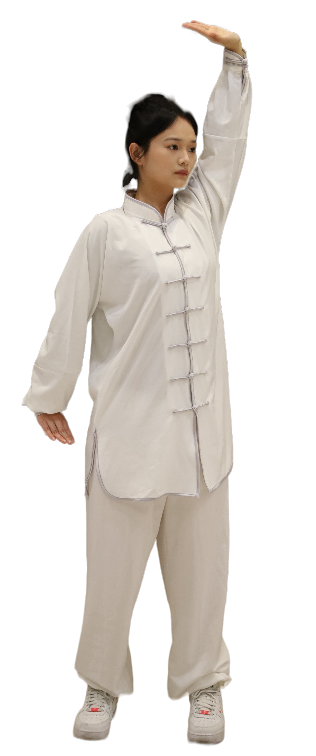  Keep the position of hips, and turn the waist to the left to the extreme. Repeat the movements of Baduanjin to raise one arm and lower the other, stretching as far as possible and staying for a breathing period. Then return to the starting position. Repeat this movement on the other side. | 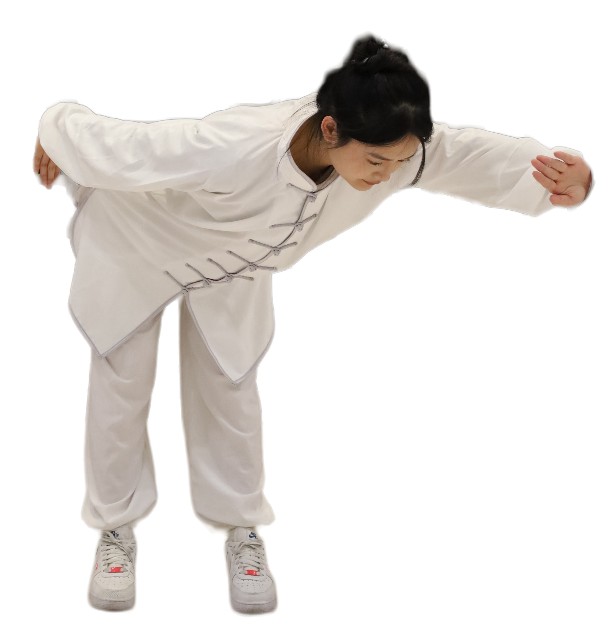  Keep the position of the hips and turn the waist to the left to the extreme. Repeat the movements of Baduanjin to raise one arm and lower the other, stretching as far as possible and bending to the left side by 90°, staying for a breathing period. Then return to the starting position. Repeat this movement on the other side. |
| **Section 4. Look Back to Prevent the Diseases and Injuries** | | |
| **Traditional Baduanjin** | **Strengthened Baduanjin (First + Second levels)** | |
| 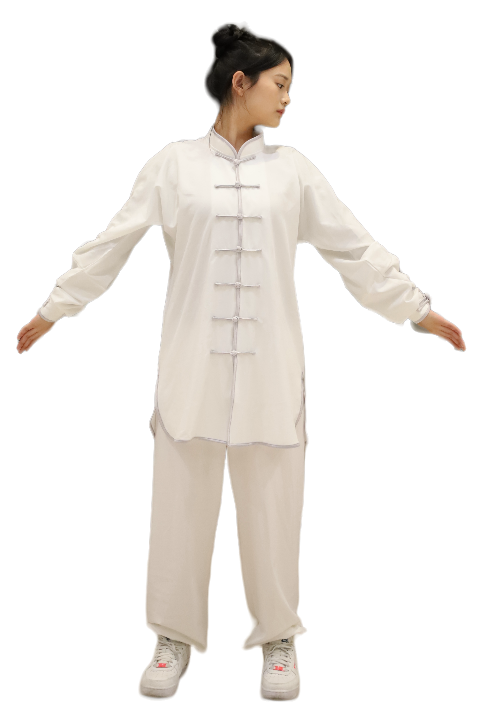  Stand with your feet shoulder-width apart, and keep your knees slightly bent. Extend your arms to the sides and fully externally rotate your arms. At the same time, turn your head to the left and look back over your shoulder, pausing briefly in this position. Bring your head back to the center, and return to the starting position. Repeat this movement on the other side. | 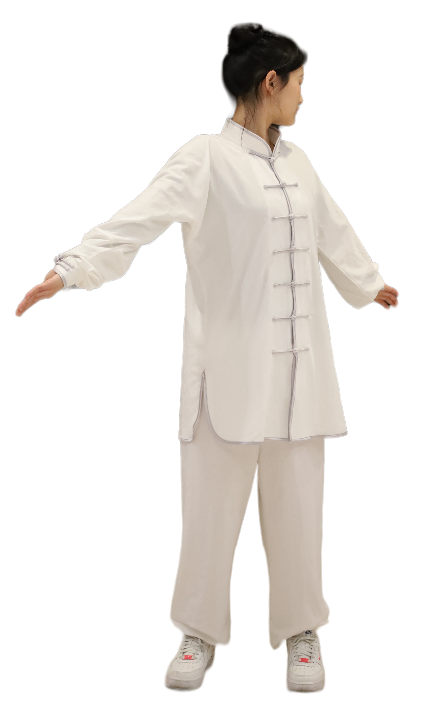  Repeat the movements of Baduanjin to extend your arms to the sides and externally rotate your arms with your head to the left and look back over your shoulder. Keep the position of hips and turn the waist to the left to the extreme, staying for a breathing period, then return to the starting position. Repeat this movement on the other side. | 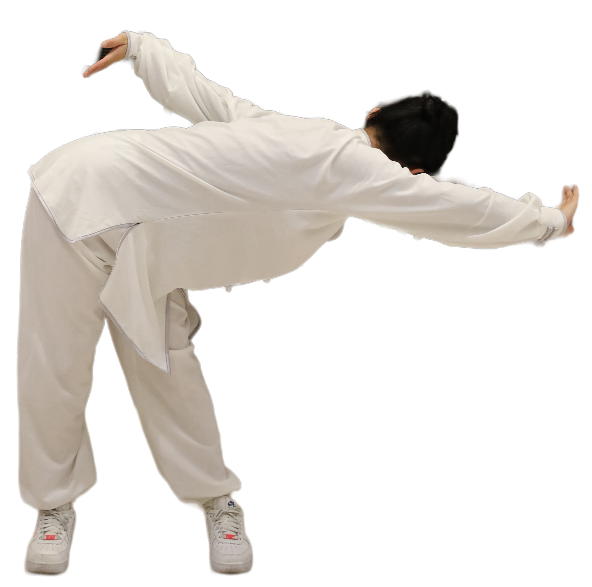  Repeat the movements of the second step to turn the waist to the left extremely. Bend forward to 90°as inward rotate the right hand, palm up, along the right ear through the palm forward. Left-hand inward rotation, bend the elbow through the waist side, palm up. Both palms should align in a straight line, stretching forward and backward, parallel to the torso. Hold this position for a breathing period, and then return to the starting position. Repeat this movement on the other side. |
| **Section 5. Sway the Head and Shake the Tail to Calm the Heart Fire** | | |
| **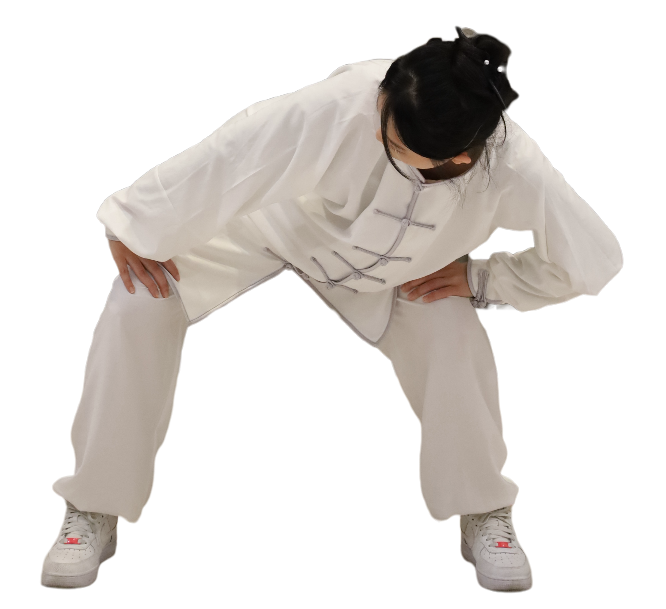**  Stand with your feet wider than shoulder-width apart and your arms hanging loosely at your sides. Raise both arms to chest height with palms facing down, then flip your arms and fall on both sides to support the knee above the legs. At the same time, bend your knees slightly, shift your weight onto one leg, shift your weight and twist your torso to the side. Then bring your weight back to the center, return to the starting position and repeat on the other side, twisting in the opposite direction. | | |
| **Section 6. Hold the Feet with Both Hands to Strengthen Waist and Kidneys** | | |
| **Traditional Baduanjin** | **Strengthened Baduanjin (First + Second levels)** | |
| 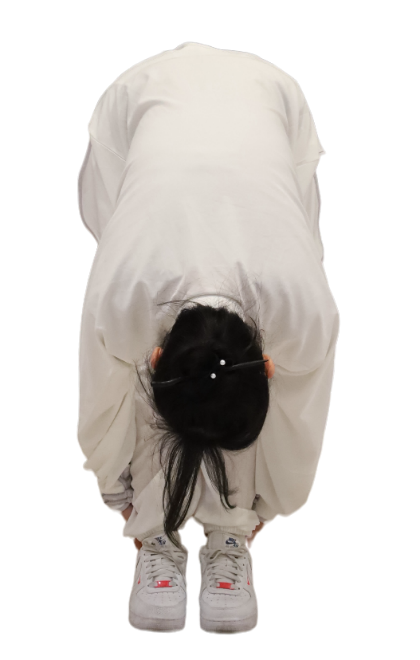  Stand with your feet shoulder-width apart and your arms at your sides. Turn the arms forward and upward over the head, then press down to your chest, thread your palms through the armpits to behind your back, palms following both sides of the spine, gliding downwards to the hips. At the same time, bend forward at the waist, keeping your knees slightly bent, and reach for your toes. If possible, grasp your ankles or feet with your hands. Then, as you straighten your legs, feel the stretch in your hamstrings. Hold for a moment, then bend your knees and return to the starting position. Repeat this movement several times. | 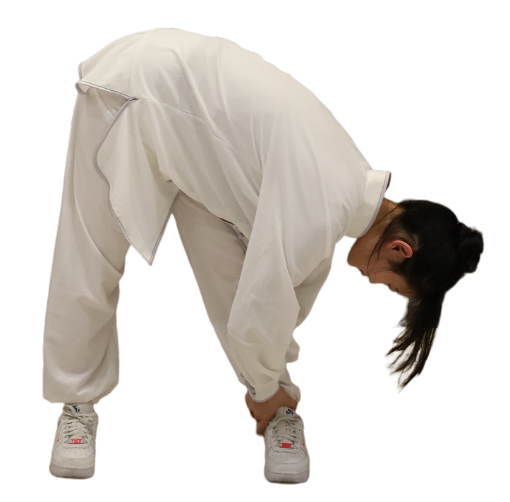  Stand with your feet shoulder-width apart and your arms at your sides. Turn the arms forward and upward over the head, keep the position of hips and turn the waist to the left for 45°. Then press down to your chest, thread your palms through the armpits to behind your back, palms following both sides of the spine, gliding downwards to the hips. At the same time, bend to the left 45°forward at the waist, keeping your knees slightly bent, and reach for your toes. Then as you straighten your legs, feel the stretch in your hamstrings. Hold for a moment, then bend your knees and return to the starting position. Repeat this movement on the other side. | 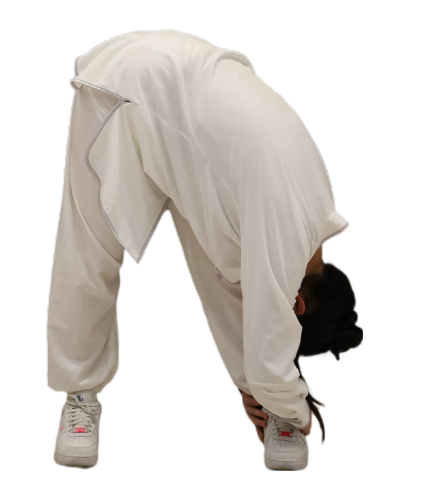  Stand with your feet shoulder-width apart and your arms at your sides. Turn the arms forward and upward over the head, keep the position of hips and turn the waist to the left for 45°. Then press down to your chest, thread your palms through the armpits to behind your back, palms following both sides of the spine, gliding downwards to the hips. At the same time, bend to the left 45°forward at the waist, keeping your knees slightly bent, and reach for your toes. Continue to run your palms down your left foot along both sides of your left leg. Gently grasp the outside of the left ankle with your right hand, gently grasp the outside of the right ankle with your left ear and right chest as close to the left thigh as possible, pausing for a breath. Then, as you straighten your legs, feel the stretch in your hamstrings. Hold for a moment, then bend your knees and return to the starting position. Repeat this movement on the other side. |
| **Section 7. Clench the Fists with Angry Eyes to Strengthen the Body** | | |
| **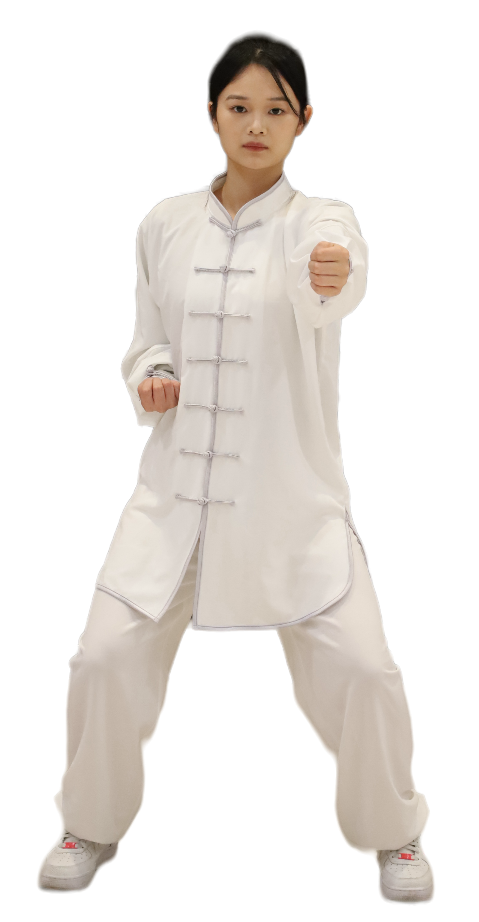**  Stand with your feet shoulder-width apart and your arms at your sides, fists closed. Punch forward with one arm, twisting your torso slightly in the direction of the punch. Be sure to keep your other arm bent and close to your body for balance. Hold for a moment, and return to the starting position. Repeat this movement on the other side. | | |
| **Section 8. Bouncing on the Toes to Cure Diseases** | | |
| **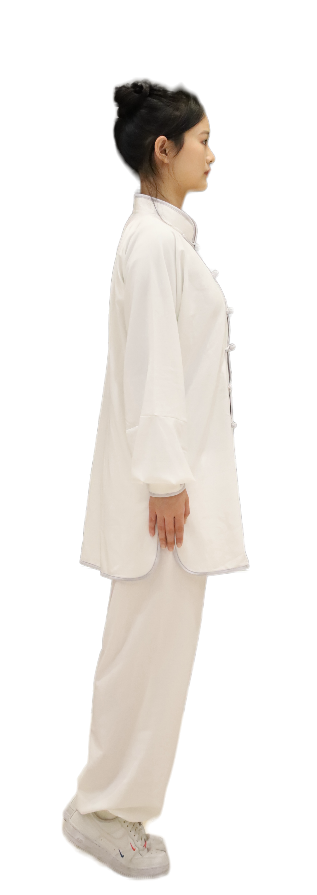**  Stand with your heels together and your arms hanging loosely at your sides. Rise onto your toes, lifting your heels off the ground. Hold for a moment and then lower your heels back down to the ground. Repeat this movement several times. | | |

**Appendix 3: Details of outcome measurements**

Each participant was asked to the hospital for the measurement of outcomes at baseline and post-intervention. During each assessment, participants were asked to complete the specified body shape measures, laboratory measures and self-reported questionnaires. All tests were administered by the trained independent assessor.

**1. Body shape measurements**

**1.1 Body mass index (BMI):** The body weight and height will be used to determine the BMI by the following calculation**,** BMI= weight/height ^2^ (kg/m^2^)

**1.2 Weight:** All participants were weighed at the same time (9:00 am to 11:00 am) before and post-intervention, and on the same scale.

**1.3 Waist circumference:** The participants stand with feet 25~30cm apart. The measuring position is the midpoint of the line connecting the anterior superior iliac crest and the lower edge of the 12th rib in the horizontal position. The measuring ruler is tightly attached to the soft tissue but cannot be compressed.

**2. Laboratory tests**

Blood samples were collected from 9:00 am to 11:00 am before and after the trial to detect glycosylated (HbAlc), fasting blood glucose (FBG), fasting insulin (FINS), triglycerides (TG), total cholesterol (TC), high-density lipoprotein cholesterol (HDL-C) and low-density lipoprotein cholesterol (LDL-C). Insulin resistance index (HOMA-IR) =[FINS(mU/L)×FPG(mmol/L)]/22.5 were calculated by the formula.

**3. Blood pressure**

Blood pressure was measured from 9:00 am to 11:00 am before and after the trial using a validated electronic sphygmomanometer. Measurements were taken in a seated position after a 5-minute rest, with the average of two consecutive readings recorded. This standardized protocol ensured accurate and reliable data collection, allowing us to assess changes in blood pressure as a secondary outcome of the study.

**4. Self-reported questionnaires**

**4.1 Fatigue Scale-14(FS-14):** was developed by Trudie Chalder of Psychological Medicine Laboratory of King's College Hospital and Queen Mary's University Hospital to evaluate the severity of fatigue symptoms and clinical efficacy, and screen fatigue cases in epidemiological studies. The fatigue scale FS-14 consists of 14 items, each of which is a fatigue-related question. Answer "Yes" or "no" according to whether the content is consistent with the actual situation of the participant. The 14 items reflect the severity of fatigue from different angles. The 14 items are divided into two categories by principal component analysis, one is physical fatigue, including 8 items (1-8), other reflects mental fatigue, including 6 items (9-14). The participant reads each item one by one or the assessor asks questions one by one to select the most appropriate situation for the participant. Except for the three items in the 10th, 13th and 14th, which are scored reversely, the answer "yes" is scored as 0, and the answer "no" is scored as 1, while the other 11 items are scored positively, that is, the answer "yes" is scored as 1, and the answer "no" is scored as 0. The highest physical fatigue score is 8, the highest mental fatigue score is 6, and the highest total score is 14. The higher the score, the more serious the fatigue is.

**4.2 The Generalized Anxiety Disorder Assessment (GAD-7):** is a self-administered 7-item measure of symptoms of a generalized anxiety disorder (GAD). The questionnaire asks participants to rate the frequency of nine symptoms of GAD within the last 2 weeks on a scale from “not at all” to “nearly every day” (scored 0–3 with a total score ranging from 0 to 21). Total scores can be interpreted of “minimal” (0–4), “mild” ([5](https://www.ncbi.nlm.nih.gov/pmc/articles/PMC10227518/#ref5)–[9](https://www.ncbi.nlm.nih.gov/pmc/articles/PMC10227518/#ref9)), “moderate” ([10](https://www.ncbi.nlm.nih.gov/pmc/articles/PMC10227518/#ref10)–[14](https://www.ncbi.nlm.nih.gov/pmc/articles/PMC10227518/#ref14)), or “severe” ([15](https://www.ncbi.nlm.nih.gov/pmc/articles/PMC10227518/#ref15)–[21](https://www.ncbi.nlm.nih.gov/pmc/articles/PMC10227518/#ref21)) anxiety.

**4.3 The Patient Health Questionnaire (PHQ-9):** is a self-administered questionnaire with 9 items, measuring the presence and severity of depressive symptoms, such as feeling down, depressed, hopeless or having little interest or pleasure in doing things. Subjects are required to rate the frequency of nine symptoms of depression on a scale from “not at all (0)” to “nearly every day ([3](https://www.ncbi.nlm.nih.gov/pmc/articles/PMC10227518/#ref3))” for the past 2 weeks. Total scores can range from 0 to 27. Depression is indicative of “mild” (scores 5–9), “moderate” (scores 10–14), “moderately severe” (scores 15–19), or “severe” depression (>20).

**4.4 The Pittsburgh Sleep Quality Index** (PSQI): is a self-rated questionnaire which assesses sleep quality and disturbances over a 1-month time interval. The scale contains 19 items across 7 factors: subjective sleep quality, sleep latency, sleep duration, habitual sleep efficiency, sleep disturbances, use of sleeping medication, and daytime dysfunction. Each factor is scored from 0 to 3, with a total score of 0 to 21. The sum of scores for these seven components yields one global score. A higher score indicates poorer sleep quality.

**4.5 Dampness syndrome scale:** The dampness syndrome scale is a TCM dampness syndrome evaluation scale composed of 30 items. Each item has 5 response options: not at all, light, mild, severe and extremely severe, among which not at all is recorded as 0 points, light is recorded as 1 point, mild is recorded as 2 points, severe is recorded as 3 points and extremely severe is recorded as 4 points. If the final score is ≥15 can be determined as having dampness syndrome.
